# Field-Reassortment of Bluetongue Virus Illustrates Plasticity of Virus Associated Phenotypic Traits in the Arthropod Vector and Mammalian Host *In Vivo*

**DOI:** 10.1128/jvi.00531-22

**Published:** 2022-06-21

**Authors:** Christopher Sanders, Eva Veronesi, Paulina Rajko-Nenow, Peter Paul Clement Mertens, Carrie Batten, Simon Gubbins, Simon Carpenter, Karin Darpel

**Affiliations:** a The Pirbright Institute, Pirbright, Surrey, United Kingdom; b National Centre for Vector Entomology, Institute of Parasitology, Vetsuisse Faculty, University of Zürich, Zürich, Switzerland; c School for Veterinary Medicine and Science, University of Nottingham, Sutton Bonington, Leicestershire, United Kingdom; St. Jude Children's Research Hospital

**Keywords:** orbivirus, reassortant, bluetongue virus, emerging diseases, diptera, ruminant, orbiviruses

## Abstract

Segmented RNA viruses are a taxonomically diverse group that can infect plant, wildlife, livestock and human hosts. A shared feature of these viruses is the ability to exchange genome segments during coinfection of a host by a process termed “reassortment.” Reassortment enables rapid evolutionary change, but where transmission involves a biological arthropod vector, this change is constrained by the selection pressures imposed by the requirement for replication in two evolutionarily distant hosts. In this study, we use an *in vivo*, host-arbovirus-vector model to investigate the impact of reassortment on two phenotypic traits, virus infection rate in the vector and virulence in the host. Bluetongue virus (BTV) (*Reoviridae*) is the causative agent of bluetongue (BT), an economically important disease of domestic and wild ruminants and deer. The genome of BTV comprises 10 linear segments of dsRNA, and the virus is transmitted between ruminants by *Culicoides* biting midges (Diptera: Ceratopogonidae). Five strains of BTV representing three serotypes (BTV-1, BTV-4, and BTV-8) were isolated from naturally infected ruminants in Europe and ancestral/reassortant lineage status assigned through full genome sequencing. Each strain was then assessed in parallel for the ability to replicate in vector *Culicoides* and to cause BT in sheep. Our results demonstrate that two reassortment strains, which themselves became established in the field, had obtained high replication ability in *C. sonorensis* from one of the ancestral virus strains, which allowed inferences of the genome segments conferring this phenotypic trait.

**IMPORTANCE** Reassortment between virus strains can lead to major shifts in the transmission parameters and virulence of segmented RNA viruses, with consequences for spread, persistence, and impact. The ability of these pathogens to adapt rapidly to their environment through this mechanism presents a major challenge in defining the conditions under which emergence can occur. Utilizing a representative mammalian host–insect vector infection and transmission model, we provide direct evidence of this phenomenon in closely related ancestral and reassortant strains of BTV. Our results demonstrate that efficient infection of *Culicoides* observed for one of three ancestral BTV strains was also evident in two reassortant strains that had subsequently emerged in the same ecosystem.

## INTRODUCTION

Segmented RNA viruses include a diverse array of species classified across 11 taxonomic families that infect a wide range of hosts that include plants, animals, fungi, bacteria and marine protists (1). A key feature of segmented RNA viruses is their ability to exchange complete segments of RNA during coinfection of a single host cell by two or more virus strains, producing hybrid progeny. This form of recombination is termed “reassortment.” In the case of segmented RNA arboviruses (arthropod-borne viruses), selection of reassortant strains with advantageous phenotypic traits can occur through replication bottlenecks within both the host and biological vector of the virus. When compared to genetic drift through mutation, reassortment can lead to more rapid changes in the phenotypic characteristics of progeny viruses and can lead to an increased transmissibility (2), increased pathogenicity (3, 4) and the potential for avirulent vaccine strains to revert to virulence in the field (5, 6).

Bluetongue virus (BTV) (*Reoviridae*) is the causative agent of bluetongue (BT), an economically important disease of domestic and wild ruminants (7). The virus is primarily spread between ruminants by *Culicoides* biting midges that act as biological vectors (8, 9). Severe clinical signs of bluetongue (BT) are most commonly observed in specific breeds of sheep (7) and are characterized by injury to the vascular and lymphatic endothelium. This can result in hemorrhage and vascular leakage that in acute cases result in fever, edema, coronitis, oral and nasal erosion, cyanosis of the tongue, and death (7, 10, 11). Cattle typically show only mild clinical signs of BT following infection, but are important reservoirs of the virus and recent outbreaks in naive populations have documented more severe clinical signs in this species, caused by specific BTV strains (12, 13).

Since the turn of the century, there has been an unprecedented shift in the epidemiology of BTV in Europe, involving the incursion of multiple strains into regions with no recorded history of transmission (8, 14). These epidemics have persisted in some cases, and BTV has become endemic in several European countries, with major consequences for livestock production and trade (15, 16). Full-genome sequencing of BTV has demonstrated that reassortment occurs at a high frequency in the field (6, 17, 18), and this has been highlighted as a potential driver of virus emergence and spread in the region (6). BTV has a linear dsRNA genome consisting of 10 segments encoding seven structural (VP1-7) and at least four nonstructural (NS1-4) proteins (19, 20).

Under experimental conditions, reassortment of genome segments between BTV strains during coinfection has been reported in *Culicoides* biting midges, ruminant hosts, and cell cultures where strains have been introduced simultaneously (21–24). Arbovirus species and strain is also known to influence vector competence (25), and reassortment has been used to generate viral strains that express different levels of infection rate in *Culicoides* vectors in the laboratory (26). The proportion of a vector population able to become fully infected with BTV following oral exposure has been demonstrated to be under a combination of genetic and environmental control in *Culicoides* (9, 27, 28). To date, however, little is known regarding the impact of field-based reassortment of segmented RNA viruses on transmission by vectors and whether vector susceptibility to infection can be affected by this process.

Clinical severity of BTV virus strains also varies *in vivo* (7, 29–31), but the molecular basis for pathogenicity of BTV strains is poorly understood (32, 33). Pathogenicity and disease outcome appear to be highly complex and cannot be explained by the presence or absence of a single genome segment or specific combinations of segments (32–34). Reassortant bluetongue viruses generated from wild type and attenuated strains using reverse genetics demonstrated impacts on pathogenicity *in vitro* and *in vivo*, albeit with limited consistency between the different host systems used. To date, specific virulence characteristics could not be assigned to specific gene segments, and hence, no markers for BTV strain virulence exist (33, 35, 36). Field derived reassortant BTV strains may therefore demonstrate differential pathogenicity or disease outcome to that of their closely related strains or those which they share serotype. Studies with field-derived reassortant strains complement those carried out using BTV strains generated synthetically through reverse genetics, which have a far greater level of artificial selection through multiple cell passage and plaque purification (33, 37).

The aim of this study is to define infection characteristics of five BTV strains of three serotypes isolated from the Mediterranean Basin and Europe in both host and vector. We identified a history of reassortment between these strains during their cocirculation in the field, which directly enabled the opportunity to examine the impact of this process on BTV infection, replications, and virulence across the transmission cycle between sheep and *Culicoides*. This represents the first fully comparative analysis of ancestral and reassortant strain characterization under highly controlled conditions using a representative BTV–sheep–*Culicoides* transmission model.

## RESULTS

### Phylogenetic lineage of parental and reassortant strains can be traced.

**FIG 1:**
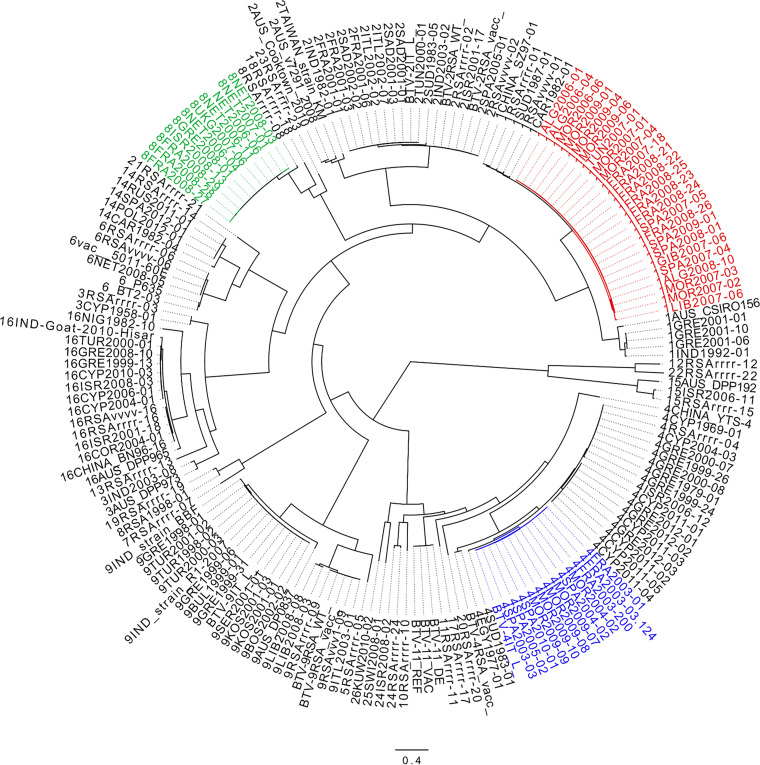
Maximum likelihood tree constructed for the VP2 coding regions of BTV using IQ-Tree software version 1.3.11.1 (64). The reliability of each tree was estimated by ultrafast bootstrap (65) analysis of 1,000 replicates. The GTR + I + G4 model of evolution was selected according to the Bayesian information criterion score calculated using the IQ-Tree software. Phylogenetic trees were visualized and rooted on the midpoint using the Figtree v1.4.4 software. BTV-1 lineage 2006–2009 is shown in red, BTV-4 lineage 2003–2010 in blue, and BTV-8 lineage 2006–2008 in green.

**FIG 2:**
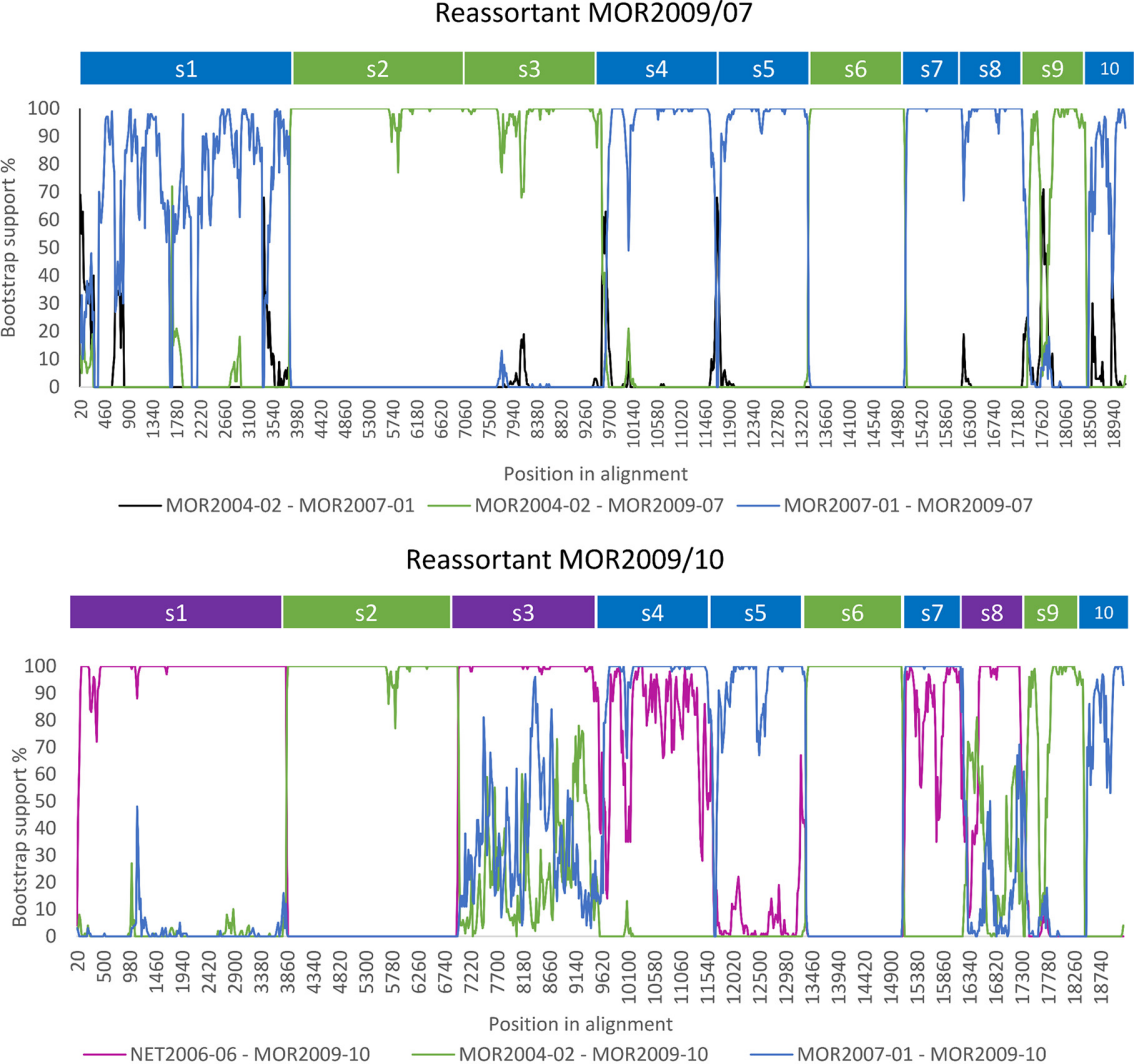
BOOTSCAN analysis of whole genome sequences of BTV-4 MOR2009/07 (top) and BTV-4 MOR2009/10 (bottom) providing evidence of genome segment reassortment. BTV-4 MOR2009/07 was identified as dual reassortment with segments 1, 4, 5, 7, 8, and 10 most closely related to BTV-1MOR2007/01 (blue) and segments 2, 3, 6, and 9 to BTV-4 MOR2004/02 (green). BTV-4 MOR2009/10 was identified as a triple reassortant, sharing segments 2, 6, and 9 from BTV-4 MOR2004/02 (green), segments 4, 5, 7, and 10 from BTV-1 MOR2007/01 (blue), and segments 1, 3, and 8 from BTV-8 NET2006/06 (magenta).

The genetic relationship between BTV strains used in the study (Table 1) was defined using sequence comparison within segments (Table 2), phylogenetic analyses (Fig. 1), and a range of additional detection methods (Fig. 2). Three strains were identified as ancestral (BTV-1 MOR2007/01, BTV-4 MOR2004/02, and BTV-8 NET2006/06), while two strains were identified as reassortant strains (BTV-4 MOR2009/07 and BTV-4 MOR2009/10) derived from the lineages of the three ancestral strains.

**TABLE 1:** Passage history of virus strains used during studies^*a*^

Virus strain	Reassortant status	Passage history of virus strain used for infection of C. *sonorensis* (no. of passages)	Viral titre quantified by immunofluorescence on KC cells
BTV-1 MOR2007/01	Parental	KC-*C. sonorensis* (2)	10^7.25^ TCID50/mL
BTV-4 MOR2004/02	Parental	Embryonated chicken egg (1); baby hamster kidney (4); KC-*C.* *sonorensis* (1)	10^5.75^ TCID50/mL
BTV-8 NET2006/06	Parental	KC-*C. sonorensis* (4)	10^6.75^ TCID50/mL
BTV-4 MOR2009/07	Reassortant	KC-*C. sonorensis* (2)	10^6.5^ TCID50/mL
BTV-4 MOR2009/10	Reassortant	KC-*C. sonorensis* (2)	10^6.25^ TCID50/mL

a All viruses were subjected to a single passage in KC-*C. sonorensis* prior to measurement of titer.

**TABLE 2:** Nucleotide identity (white) and amino acid similarity (gray) expressed as % between bluetongue virus (BTV) strains across all 10 genome segments

Seg	NET2006-06	MOR2007-01	MOR2004-02	MOR2009-07	MOR2009-10
Seg-1					
BTV-8 NET2006-06		99.3	99.0	99.1	99.7
BTV-1 MOR2007-01	94.5		99.6	99.7	99.3
BTV-4 MOR2004-02	94.7	98.8		99.4	99.0
BTV-4 MOR2009-07	94.5	99.8	98.7		99.2
BTV-4 MOR2009-10	99.9	94.5	94.6	94.5	
Seg-2					
BTV-8 NET2006-06		52.5	40.8	40.8	40.8
BTV-1 MOR2007-01	55.1		40.4	40.4	40.4
BTV-4 MOR2004-02	48.6	48.7		99.4	99.2
BTV-4 MOR2009-07	48.6	48.5	99.6		99.7
BTV-4 MOR2009-10	48.5	48.5	99.4	99.8	
Seg-3					
BTV-8 NET2006-06		99.6	99.7	99.8	100.0
BTV-1 MOR2007-01	95.4		99.6	99.7	99.6
BTV-4 MOR2004-02	95.0	95.0		99.8	99.7
BTV-4 MOR2009-07	94.6	94.6	99.2		99.8
BTV-4 MOR2009-10	99.6	95.1	94.8	94.7	
Seg-4					
BTV-8 NET2006-06		99.2	98.4	99.2	99.2
BTV-1 MOR2007-01	98.2		98.9	100.0	100.0
BTV-4 MOR2004-02	95.7	95.9		98.9	98.9
BTV-4 MOR2009-07	97.9	99.8	95.9		100.0
BTV-4 MOR2009-10	98.0	99.9	95.9	99.9	
Seg-5					
BTV-8 NET2006-06		99.6	99.4	99.6	99.6
BTV-1 MOR2007-01	94.4		99.4	100.0	100.0
BTV-4 MOR2004-02	94.7	96.6		99.4	99.4
BTV-4 MOR2009-07	94.3	99.8	96.7		100.0
BTV-4 MOR2009-10	94.0	99.6	96.4	99.6	
Seg-6					
BTV-8 NET2006-06		84.9	78.5	78.5	78.5
BTV-1 MOR2007-01	73.4		77.9	77.9	77.9
BTV-4 MOR2004-02	69.8	69.2		99.6	99.6
BTV-4 MOR2009-07	70.0	69.2	99.5		100.0
BTV-4 MOR2009-10	69.9	69.2	99.4	99.8	
Seg-7					
BTV-8 NET2006-06		100.0	99.7	100.0	100.0
BTV-1 MOR2007-01	97.0		99.7	100.0	100.0
BTV-4 MOR2004-02	94.2	94.0		99.7	99.7
BTV-4 MOR2009-07	97.1	99.9	94.1		100.0
BTV-4 MOR2009-10	97.1	99.9	94.1	100.0	
Seg-8					
BTV-8 NET2006-06		97.7	98.3	97.7	99.4
BTV-1 MOR2007-01	95.6		99.4	100.0	97.7
BTV-4 MOR2004-02	96.0	95.9		99.4	98.3
BTV-4 MOR2009-07	95.5	99.8	95.8		97.7
BTV-4 MOR2009-10	99.2	95.2	95.6	95.2	
Seg-9					
BTV-8 NET2006-06		96.3	95.7	95.7	95.4
BTV-1 MOR2007-01	96.3		96.9	97.5	97.2
BTV-4 MOR2004-02	96.2	97.2		98.7	98.4
BTV-4 MOR2009-07	96.2	97.0	99.1		99.6
BTV-4 MOR2009-10	96.1	96.9	99.1	99.9	
Seg-10					
BTV-8 NET2006-06		95.1	94.7	95.1	95.1
BTV-1 MOR2007-01	83.3		99.5	100.0	100.0
BTV-4 MOR2004-02	83.2	98.3		99.5	99.5
BTV-4 MOR2009-07	83.6	99.2	97.7		100.0
BTV-4 MOR2009-10	83.5	99.4	97.9	99.8	

Two of the ancestral strains (BTV-1 MOR2007/01 and BTV-4 MOR2004/02) were isolated from samples collected in Morocco, while the third ancestral strain (BTV-8 NET2006/06) was isolated from a sample collected in the Netherlands. Strains of this BTV-8 lineage spread throughout Northern Europe and the Mediterranean Basin between 2007 and 2010, therefore cocirculating in the same ecosystem as the other BTV strains used in this study. The field-derived reassortant strains, BTV-4 MOR2009/07 and BTV-4 MOR2009/10, were also isolated from samples collected in Morocco. The beginning and end recombination breakpoints (shown in Fig. 2) corresponded with the segment position in the sequence alignment and are for Seg-1 (1-3944), Seg-2 (3945-6910), Seg-3 (6911-9682), Seg-4 (9683-11663), Seg-5 (11664-13441), Seg-6 (13442-15079), Seg-7 (15080-16235), Seg-8 (16236-17360), Seg-9 (17361-18411), and Seg-10 (18412-19233). Both reassortant strains had Seg-2 derived from BTV-4 MOR2004/02, and therefore both belonged to the same genotype/serotype (Fig. 1).

The detection of the reassortant BTV-4 MOR2009/07 was supported by seven different detection methods within the recombination detection program: RDP (1.00 × 10^−10^), GENECONV (7.42 × 10^−110^), Bootscan (6.62 × 10^−131^), Maximum Chi Square (4.02 × 10^−28^), CHIMAERA (2.03 × 10^−29^), SISCAN (5.26 × 10^−31^), and 3SEQ (3.22 × 10^−100^). BTV-1 MOR2007/01 (Seg-1, 4, 5, 7, 8, and 10) BTV-4 MOR2004/02 (Seg-2, 3, 6, and 9) sequences were identified as the primary and secondary ancestral strains, respectively (Fig. 2). The detection of the second reassortant (BTV-4 MOR2009/10) was supported by six different detection methods: GENECONV (4.55 × 10^−136^), Bootscan (4.64 × 10^−4^), Maximum Chi Square (4.18 × 10^−27^), CHIMAERA (5.92 × 10^−36^), SISCAN (1.42 × 10^−37^) and 3SEQ (1.11 × 10^−15^). BTV-4 MOR2009/10 was identified as a triple reassortant, sharing segments from BTV-4 MOR2004/02 (Seg-2, -6, and -9), BTV-1 MOR2007/01 (Seg-4, 5, 7, and 10) and BTV-8 NET2006/06 (Seg-1, 3, and 8) strains (Fig. 2, Table 2). It is probable that the BTV-4 MOR2009/10 emerged from a single cell coinfection with two ancestral strains (BTV-4 MOR2009/07 and BTV-8 NET2006/06) rather than simultaneous coinfection with three different ancestral BTV strains (BTV-1 MOR2007/01, BTV-4 MOR2004/02, and BTV-8 NET2006/06).

As both the BTV-4 MOR2009/07 and BTV-4 MOR2009/10 reassortants were derived from the same ancestral strains, they were either identical or close to identical at the nucleotide level in Seg-2 (99.8%), -4 (99.9%), -5 (99.6%), -6 (99.8%), -7 (100%), -9 (99.9%), and -10 (99.8%). However, BTV-4 MOR2009/07 and BTV-4 MOR2009/10 differed in the remaining segments: Seg-1 (94.5%), Seg-3 (94.7%), and Seg-8 (95.2%), as these segments were more closely related to BTV-8 NET2006/06 in the triple reassortant strain BTV-4 MOR2009/10 (Table 2).

### Infection of sheep with BTV by infected *Culicoides* is highly efficient, independent of BTV strain.

**FIG 3:**
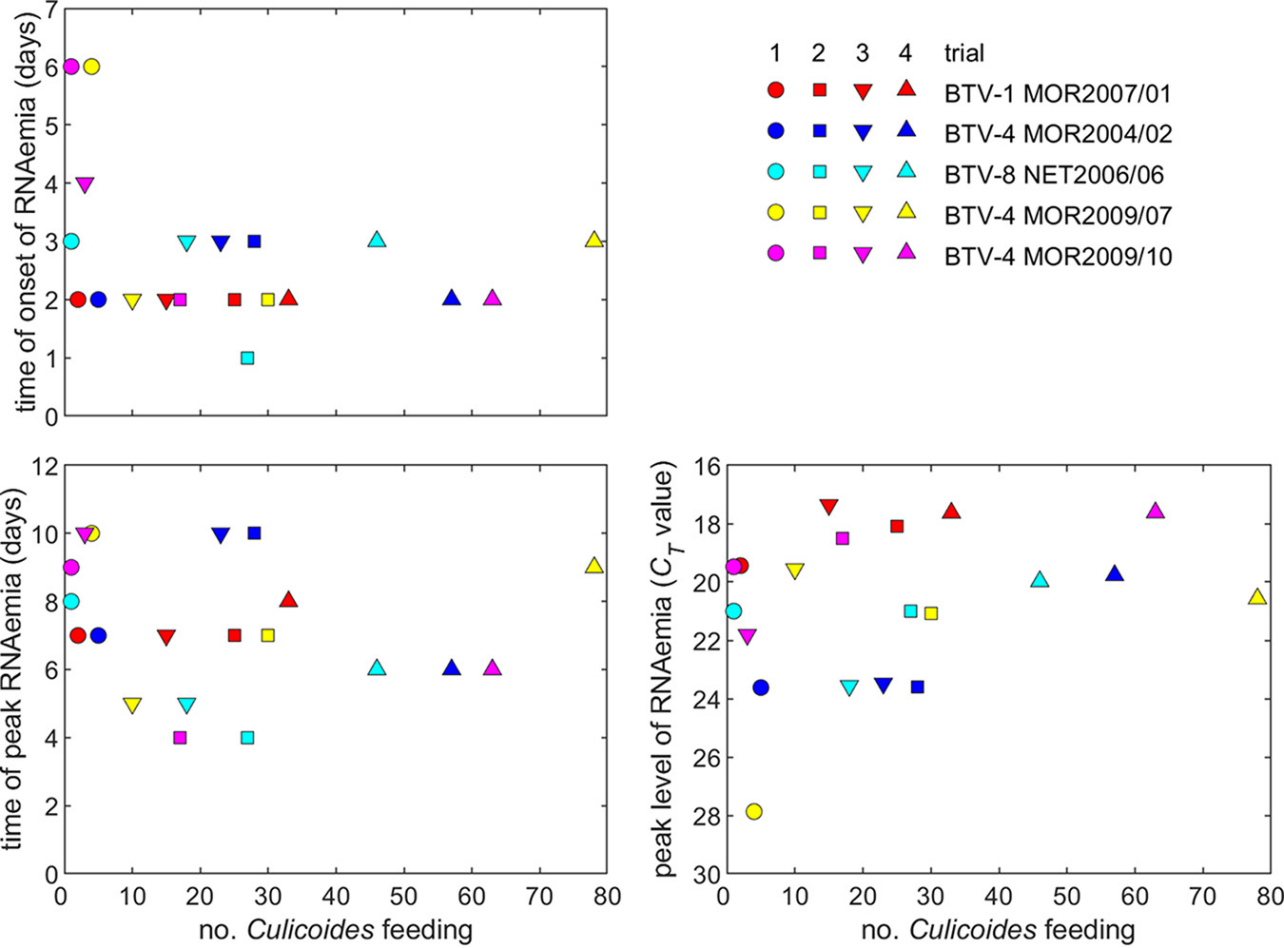
Time to onset of RNAemia, time of peak RNAemia, and level of peak RNAemia in sheep infected with different strains of bluetongue virus and their dependence on the number of inoculated *Culicoides* feeding to initiate the infection. Strains are indicated by color: BTV-1 MOR2007/01 (red), BTV-4 MOR2004/02 (blue), BTV-8 NET2006/08 (cyan), BTV-4 MOR2009/07 (yellow), and BTV-4 MOR2009/10 (magenta). Symbols indicate the sheep from each trial (trial 1, circles; trial 2, squares; trial 3, down triangles; trial 4, up triangles).

**FIG 4:**
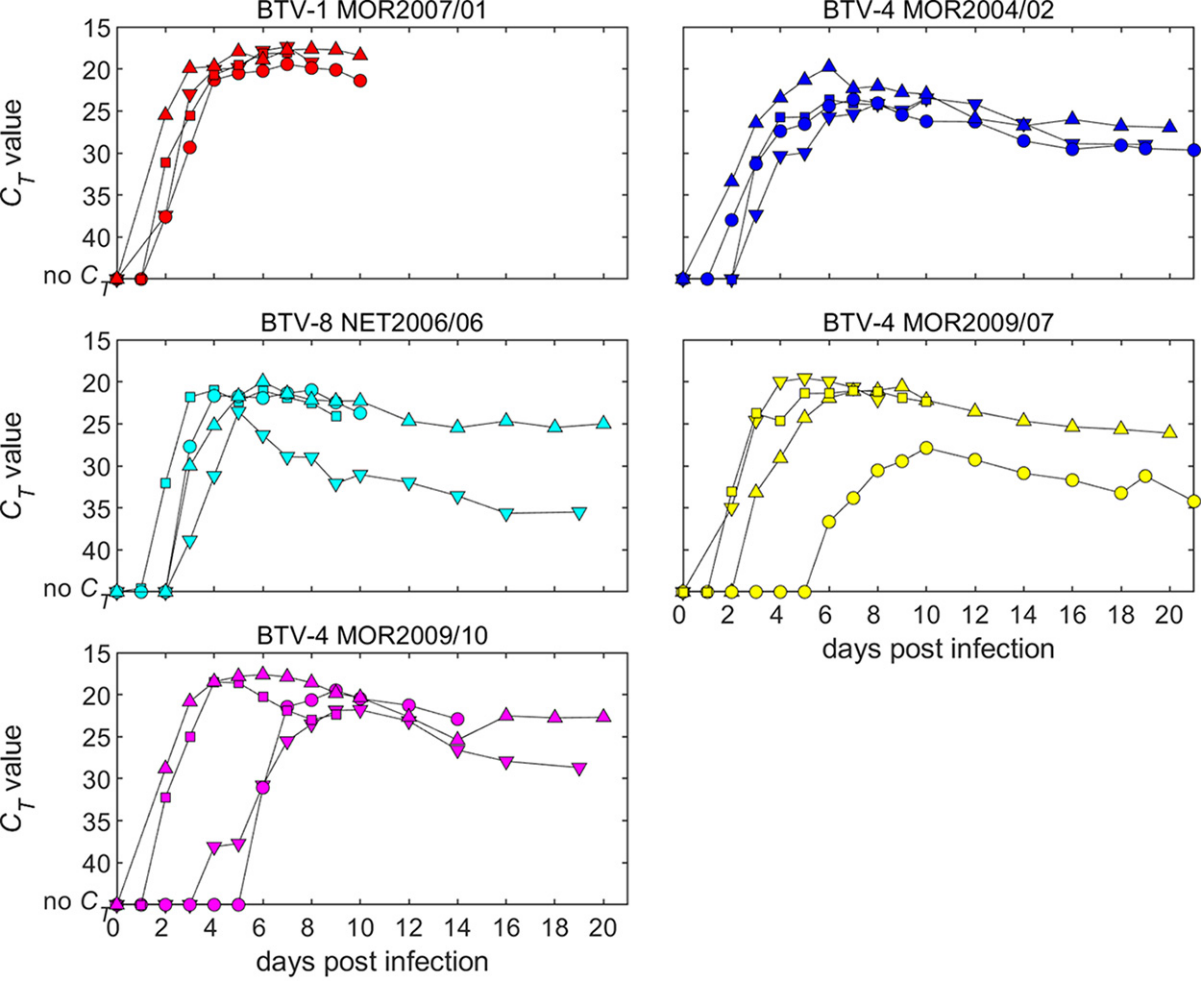
RNAemia in sheep infected with one of five strains of bluetongue virus. Each plot shows the changes in *C_T_* value over time for individual sheep. The symbols indicate the sheep from each trial infected with each strain (trial 1, circles; trial 2, squares; trial 3, down triangles; trial 4, up triangles).

A total of 2,762 *C. sonorensis* were intrathoracically inoculated (ITI) with the 5 strains of BTV, of which 1,121 (40.6%) survived the incubation period and 486 (17.6%) successfully blood fed on sheep (Table 3). All sheep in all replicates were successfully infected with BTV despite ≤10 infected *C. sonorensis* taking a blood meal in 6 of 20 infection attempts and in two infection attempts where only one individual *C. sonorensis* took a blood meal from the sheep (Table 3). All five strains of BTV were successfully transmitted on at least one occasion from the bites of 5 or fewer infected *C. sonorensis* during the trials (Fig. 3; Fig. 4).

**TABLE 3:** Inoculation and feeding success of *Culicoides sonorensis* infected with five strains of BTV across four cohorts of sheep infection studies

Virus	Exptl cohort	No. inoculated	No. surviving incubation	No. blood fed (% of survived)
BTV-1 MOR2007/01 (Parental)	1	137	34	2 (5.9)
	2	141	98	25 (25.5)
	3	100	27	15 (55.6)
	4	155	47	33 (70.2)
BTV-4 MOR2004/02 (Parental)	1	129	31	5 (16.1)
	2	148	73	28 (38.4)
	3	120	48	23 (47.9)
	4	167	109	57 (52.3)
BTV-8 NET2006/06 (Parental)	1	115	23	1 (4.3)
	2	141	84	27 (32.1)
	3	105	28	18 (64.3)
	4	175	80	46 (57.5)
BTV-4 MOR2009/07 (Reassortant)	1	168	41	4 (9.8)
	2	141	57	30 (52.6)
	3	109	42	10 (23.8)
	4	167	112	78 (69.6)
BTV-4 MOR2009/10 (Reassortant)	1	160	14	1 (7.1)
	2	131	63	17 (27.0)
	3	94	9	3 (33.3)
	4	159	101	63 (62.4)

### Level of BTV RNAemia in sheep varies with strain and number of infected *Culicoides* feeding.

There was no significant effect of BTV strain (*P* = 0.47) or number of *Culicoides* feeding (*P* = 0.58) on the timing of peak RNAemia (Fig. 3 and 4). The level of peak RNAemia differed significantly among BTV strains (*P* = 0.009), with a higher level of RNA detected (i.e., lower *C_T_* value) for BTV-1 MOR2007/01 compared with BTV-4 MOR2004/02 (*P* = 0.02) and BTV-4 MOR2009/07 (*P* = 0.02) (Fig. 3 and 4). In addition, the level of peak RNAemia increased significantly (i.e., the *C_T_* value decreased) with the number of *Culicoides* feeding (*P* = 0.02, b = −0.05, 95% confidence interval: −0.09 to −0.01) (Fig. 3). At an individual replicate level, several sheep produced a late and slowly progressing RNAemia (Fig. 4). In these three cases, BTV RNA was not detected until 5 or 6 days postinfection. These cases involved BTV-4 MOR2009/07 transmitted by four *C. sonorensis* and two infections of BTV-4 MOR2009/10 by one and three biting midges, respectively (Table 3).

### Clinical disease in infected sheep varies with BTV strain and illustrates multifactorial causation.

**FIG 5:**
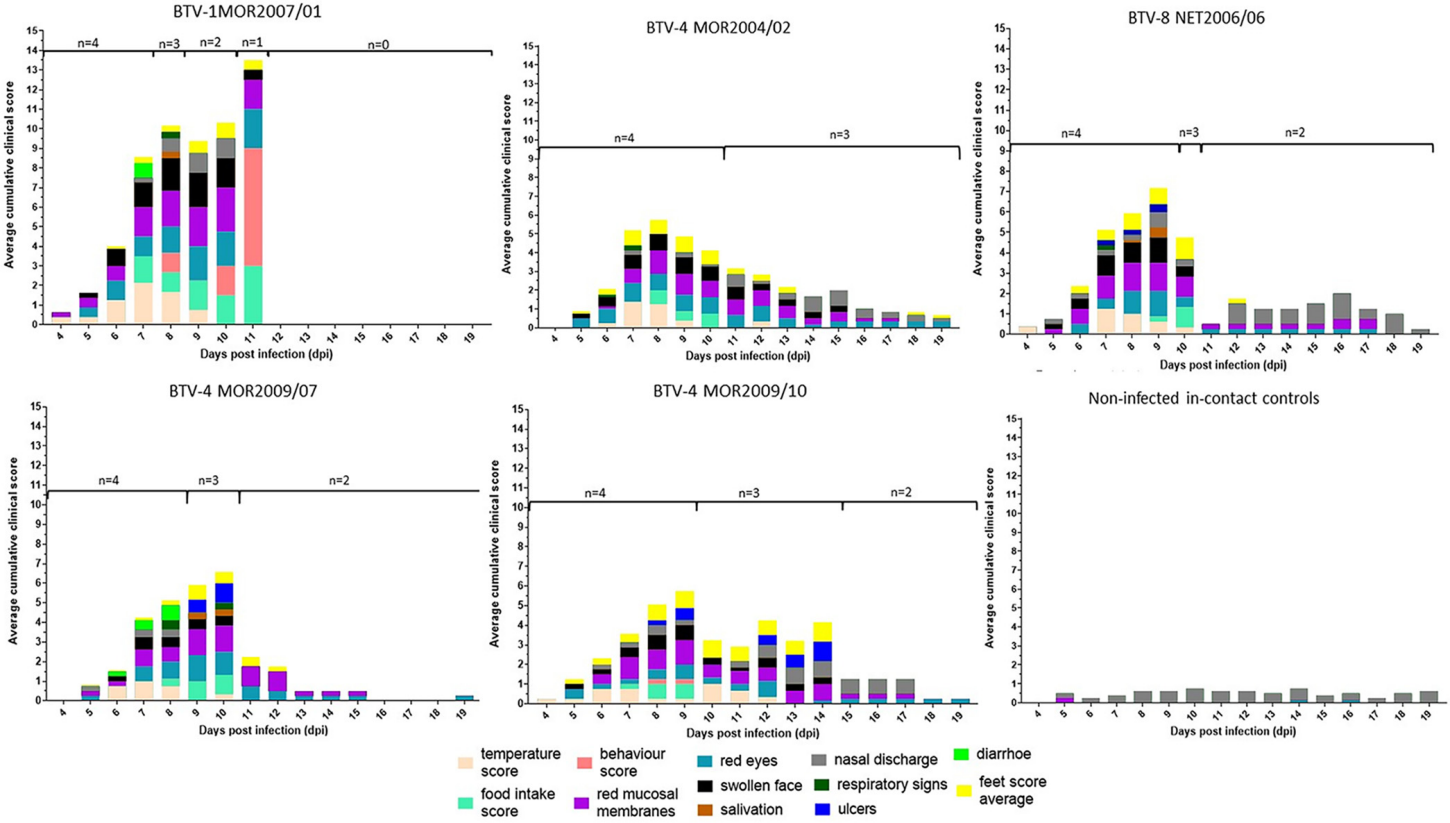
Daily accumulated average clinical score observed in sheep infected with 5 different BTV strains. Clinical scores were obtained across 12 different clinical signs routinely observed during BTV infection and visualized for each of the separate clinical signs for the time period of 4–19 dpi (see legend). The daily clinical scores recorded for each clinical sign were combined from all sheep within the group and then normalized to the sheep still present on the day to account for removal of sheep from groups at different days due to reaching predefined humane endpoints. Clinical scores for the uninfected in-contact controls were also recorded throughout to highlight potential nonspecific clinical observations.

**FIG 6:**
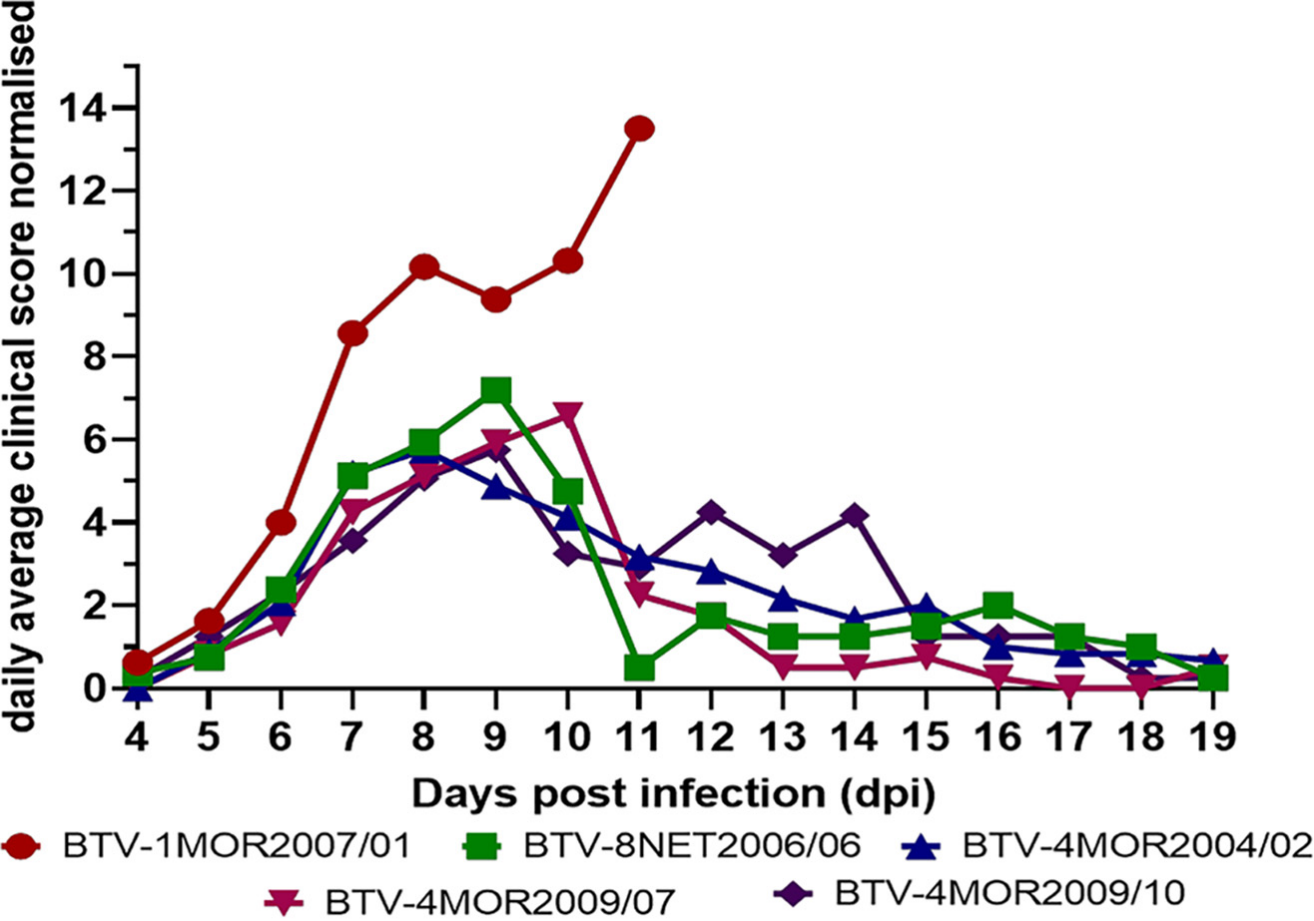
Clinical severity of 5 different BTV strains over time. The daily average normalized clinical score combined for all clinical signs visualized for the clinical period between 4 and 19 dpi for each of the 5 different BTV strains.

Infection with BTV-1 MOR2007/01 caused a consistent acute clinical disease in all four exposed sheep necessitating euthanasia between 7 and 11 days postinfection (dpi) for reaching predefined humane endpoints (Fig. 5 and 6; Tables 4 and 5). All other BTV strains exhibited within cohort variation in clinical disease signs. Three of the BTV strains used produced acute clinical disease necessitating euthanasia in two sheep and milder, chronic disease in the other two (Fig. 5 and 6; Table 5), while the ancestral strain of BTV-4 MOR2004/02 only led to acute disease in one sheep, which at postmortem showed only mild pathological signs. Although the overall severity score of the ancestral strain, BTV-4 MOR2004/02 was comparable to those of the reassortment strains BTV-4 MOR2009/07 and BTV-4 MOR2009/10, BT presented as more chronic, reflected in a higher clinical index score accumulation based on signs such reddening of eyes, nasal discharge, and feet lesions (Fig. 5; Fig. 6; Table 5).

**TABLE 4:** Clinical scoring used to assess severity of disease signs in sheep infected with BTV parental and reassortant strains

Clinical sign	Observation	Increment of score assigned (Range)
1. Redness of eyes	Severity	0.5 (0.5: mild to 3: severe)
2. Redness of mucosal membrane	Severity	0.5 (0.5: mild to 3: severe)
3. Facial edema	Swelling	0.5 (0.5: mild to 3: severe)
4. Salivation	Salivation	0.5 (0.5: mild to 3: severe)
5. Nasal discharge	Discharge	0.5 (0.5: mild to 3: severe)
6. Cough	Frequency and Severity	0.5 (0.5: mild to 3: severe)
7. Ulcers (oral and/or nasal)	Severity	0.5 (0.5: mild to 3: severe)
8. Diarrhea	Severity	0.5 (0.5: mild to 3: severe)
9. Body temp	°C	Increase of ≥1°C from resting = 1; ≥1.5°C = 1.5 and ≥2°C = 2; temp >41°C = 3
10. Behavior	General observation	Apathy and slowness = 1; Reluctance to rise and separation from flock = 2; Reluctance to rise with stimulus = 3; Refusal to rise with stimulus = 3 and humane endpoint
11. Food Intake	Consumption scored across 2 daily meals and then averaged/day	Reduced food intake = 1; avoiding concentrate but eating hay = 2; no food intake = 3
12. Feet	Determined separately for each foot and then averaged for an individual	0.5 (0.5–3 considering warmth, reddening, lesions and lameness)

**TABLE 5:** Summary of clinical severity of all five parental and reassortant BTV strains in sheep

Group	Total clinical score	Days alive all sheep 4–19 dpi (max 64 days if all survived)	Normalized severity score (total score/days alive)	Clinical observation notes
BTV-1MOR2007/01 (Parental)	142.625	24	5.94	Highly acute disease presentation, euthanasia of all sheep 7–11 dpi.
BTV-4 MOR2004/02 (Parental)	137	55	2.49	Acute clinical presentation only in one sheep; however, high accumulation of scores across chronic clinical signs (red eyes and mucosal membranes but also swollen face).
BTV-8 NET2006/06 (Parental)	122.75	45	2.73	Clearly separated into two acutely affected sheep and 2 mild but chronically affected sheep.
BTV-4 MOR2009/07 (Reassortant)	97.5	44	2.22	Clearly separated into two acutely affected sheep and 2 mild but chronically affected sheep. Lower accumulated feet score than other strains.
BTV-4 MOR2009/10 (Reassortant)	134.625	49	2.75	Clearly separated into two acutely affected sheep and 2 mild but chronically affected sheep. One of the sheep developed peak clinical disease late with feet lesions and burned muzzle (ulcers) (euthanasia 14 dpi).
Uninfected controls	31	64	0.49	Two sheep with nasal discharge for >10 days each.

**FIG 7:**
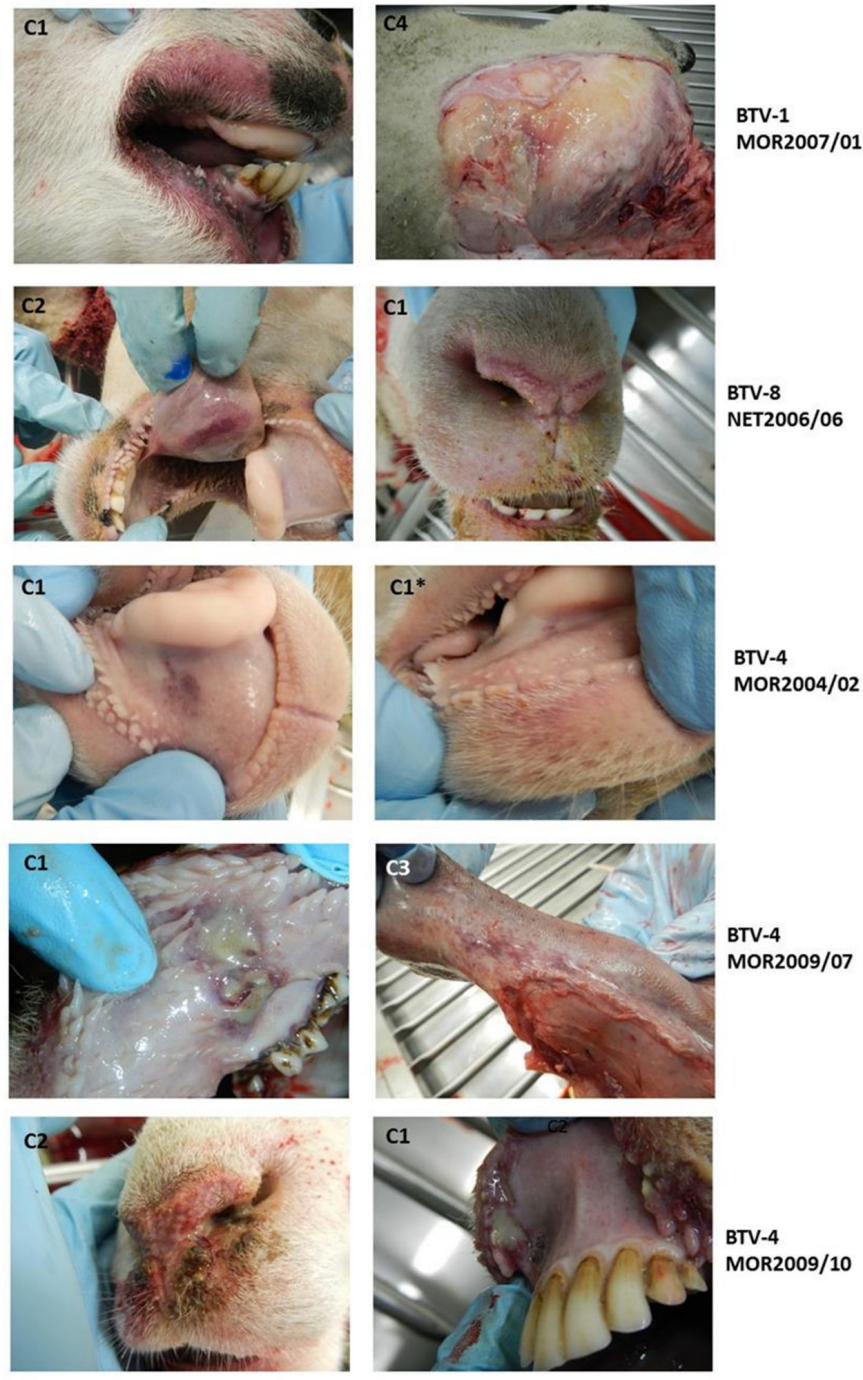
Typical pathological changes in acutely affected sheep infected with 5 different strains of BTV. Pathological signs observed in sheep euthanized for reaching humane endpoints between 7 and 14 days postinfection. The cohort of the sheep is given in each panel. BTV-1 MOR2007/01 caused wide-ranging hemorrhages in the oral and nasal mucosa, but also skeletal muscles, as well as significant subcutaneous facial and systemic edema (4/4 sheep). BTV-8 NET2006/06 caused moderate hemorrhagic lesions to the oral and nasal mucosa and tongue (2/4 sheep). BTV4 MOR2004/02 only caused mild hemorrhagic lesions of the oral mucosa (1/4 sheep; both pictures of the same sheep). Both BTV-4 reassortant strains (BTV4 MOR 2009/07 and BTV-4 MOR 2009/10) caused oral and nasal hemorrhagic lesions exceeding those of the BTV-4 and BTV-8 parental strains, but less severe than the BTV-1 parental strain (2/4 sheep for each strain). These two viral strains caused significant oral or nasal ulcerations in the affected sheep that were more severe than those seen for any parental BTV strain.

At postmortem all four sheep infected with BTV-1 MOR2007/01 demonstrated systemic hemorrhages and edema that were not only confined to the oral and nasal cavity, but also generalized through the subcutaneous layer and skeletal muscles (Fig. 7). These lesions were less pronounced in BTV-8 NET2006/04 and further reduced in BTV-4 MOR2004/02. Three of the four acutely affected sheep infected with either of the reassortant strains BTV-4 MOR2009/07 or BTV-4 MOR2009/10 developed overt ulceration, either of the oral or labial mucosa, or of the muzzle. Only one sheep from the BTV-8 NET2006/04 cohort developed mild ulceration among all the ancestral strains (Fig. 7). The acutely affected sheep infected with either reassortant strain also developed significant hemorrhages in the oral/nasal cavities that were slightly less severe than in BTV-1 MOR2007/01, but exceeding those observed for BTV-4 MOR2004/02 and BTV-8 NET2006/04. All sheep that had exhibited mild or moderate clinical disease and survived to the study end at 19/20 dpi only demonstrated mild pathological signs of BTV infection at postmortem, independent of the BTV strain.

### BTV infection efficiency in *Culicoides* varies significantly according to virus strain and can change rapidly via reassortment in the field.

**FIG 8:**
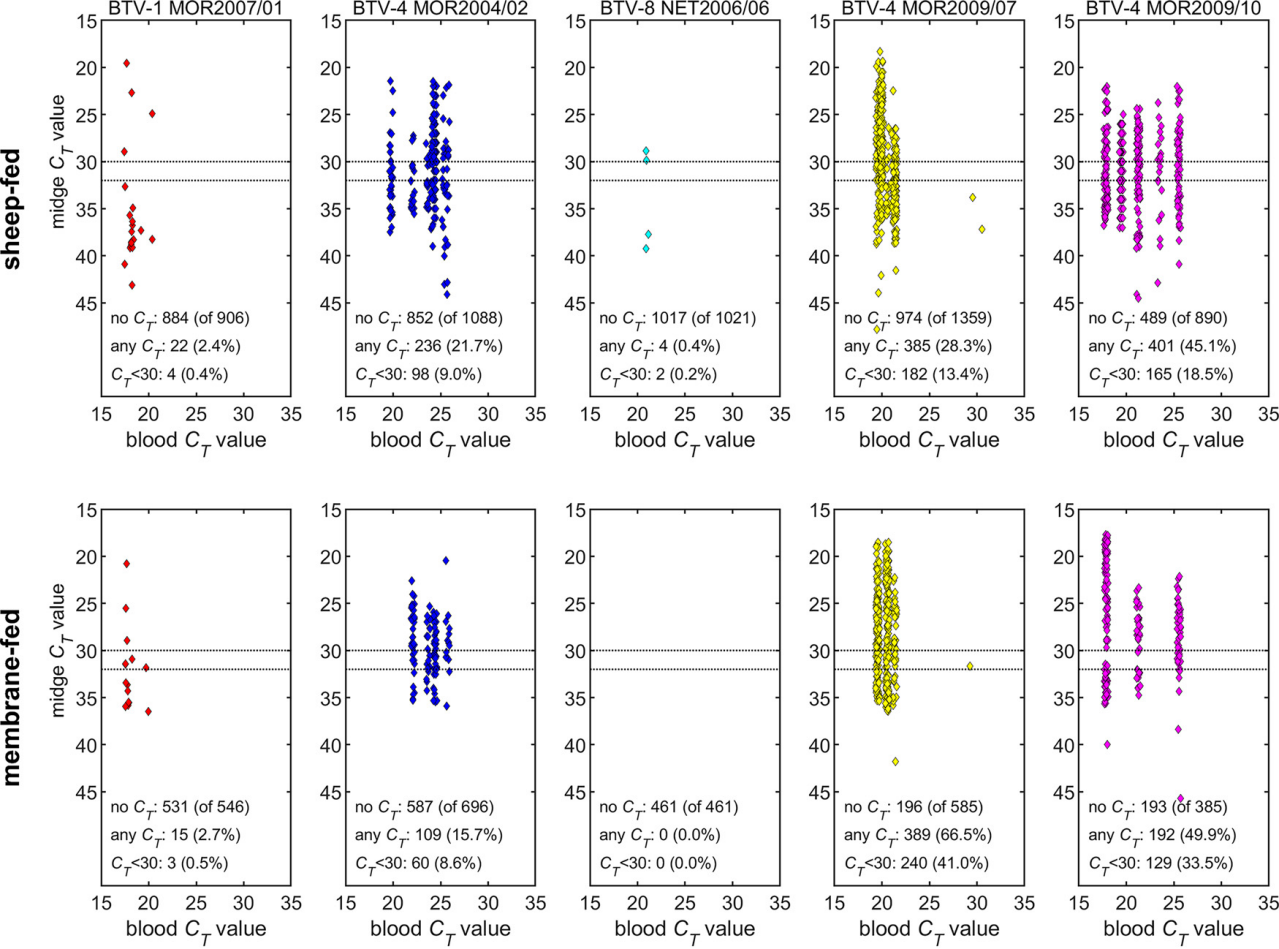
Observed *C_T_* values for five strains of bluetongue virus in *Culicoides sonorensis* following blood feeding and incubation for 8 days at 25°C. Each plot shows the dependence of the *C_T_* value on feeding route (sheep-fed, top row; membrane-fed, bottom row) and the *C_T_* value of the infected blood meal. The dotted lines indicate *C_T_* values that correspond to a possible (*C_T_* < 32) and probable (*C_T_* < 30) fully transmissible infection in a previous study of BTV infection in *C. sonorensis* (62).

**FIG 9:**
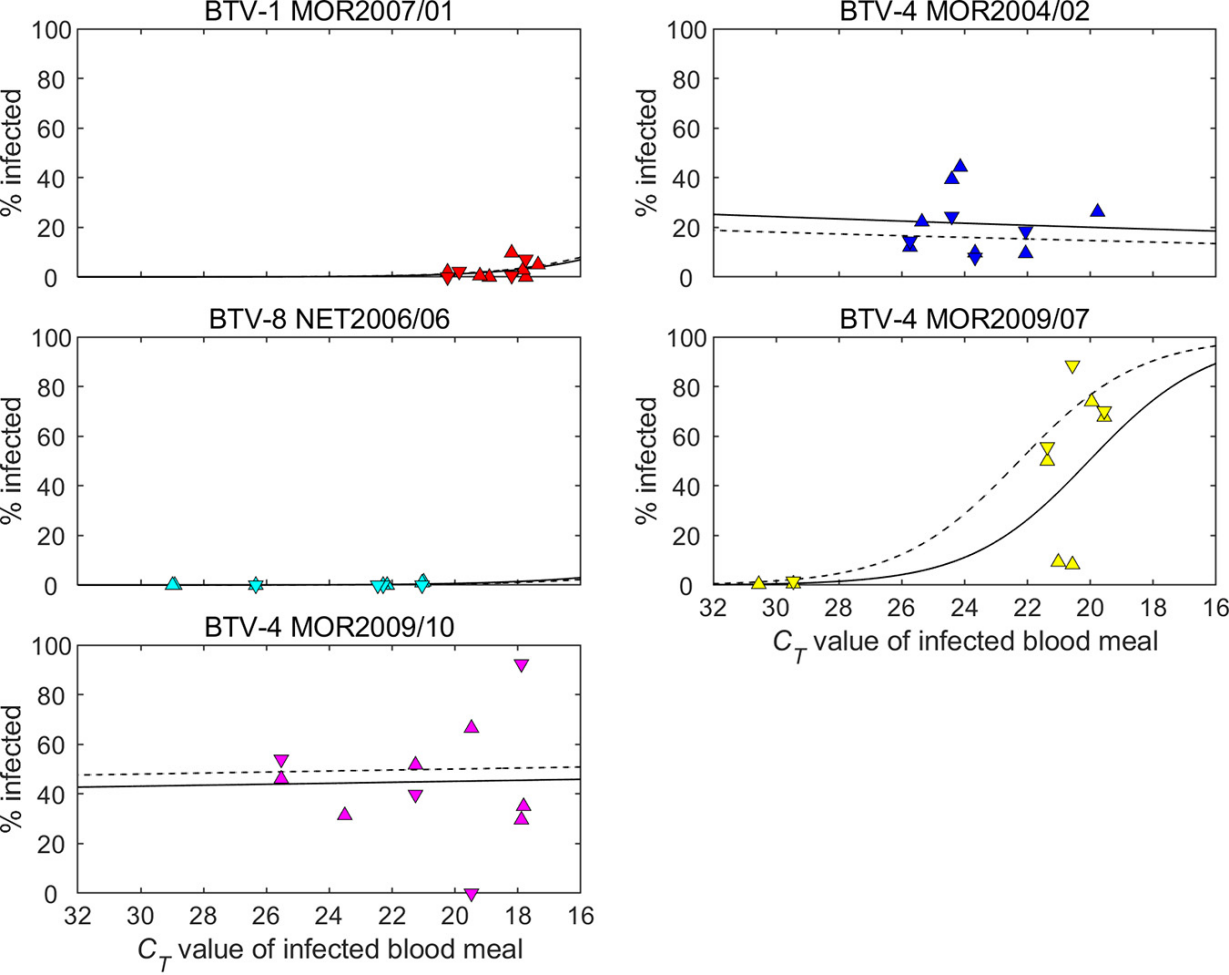
Proportion (%) of *Culicoides sonorensis* infected after feeding on blood containing one of five strains of bluetongue virus and its dependence on feeding route and blood meal *C_T_* value. Each plot shows the posterior median for the proportion infected following feeding on a sheep (solid black line) or via a membrane (dashed black line). The symbols show the observed proportions of infected midges at each *C_T_* value following feeding on a sheep (up triangles) or via a membrane (down triangles).

A total of 5,263 out of 6,457 (81.5%) female *C. sonorensis* that were fed directly on viraemic sheep survived 8 days of incubation across the four replicates (Fig. 8). In addition, a total of 2,673 out of 4,943 (54.1%) female *C. sonorensis* were successfully membrane-fed on matched blood from the viraemic sheep and survived the period of incubation (Fig. 8). The proportion of *Culicoides* in which BTV RNA was detected varied significantly between strains tested (Fig. 8). BTV-1 MOR2007/01 (2.4%) and BTV-8 NET2006/04 (0.4%) infected only a small proportion of adult *C. sonorensis*, while the third ancestral strain, BTV-4 MOR2004/02, demonstrated a significantly greater replication efficiency, resulting in an infection rate of 21.7%. Both reassortant strains BTV-4 MOR2009/07 (28.3%) and BTV-4 MOR2009/10 (45.1%) retained this efficient infectivity rate in *C. sonorensis*. The effect of blood-feeding route (sheep versus membrane-based system) differed among the strains, but broadly recapitulated the clear difference in infectivity between BTV-1 MOR2007/01 and BTV-8 NET2006/04 in comparison to the three other BTV strains (Fig. 8 and 9; Table 6) in adult *C. sonorensis*. In BTV-4 MOR2004/02, membrane feeding was associated with decreased viral replication in the insect vector compared with feeding on an infected sheep, while for BTV-4 MOR2009/07 it was associated with an increase. For the other three strains, there was no significant difference in vector infection efficiency between the two feeding routes (Fig. 9; Table 6).

**TABLE 6:** Odds ratios showing the effect of blood meal *C_T_* value and feeding route on viral infection of *C. sonorensis* for five strains of bluetongue virus

Strain	Blood meal *C_T_* value^*a*^			Feeding route^*b*^		
	Estimate	95% credible limit		Estimate	95% credible limit	
		Lower	Upper		Lower	Upper
BTV-1 MOR2007/01	**0.64**	0.43	0.88	1.15	0.63	2.10
BTV-4 MOR2004/02	1.02	0.96	1.10	**0.68**	0.53	0.88
BTV-8 NET2006/06	0.69	0.33	1.01	0.78	0.12	2.31
BTV-4 MOR2009/07	**0.59**	0.54	0.64	**3.19**	2.52	4.08
BTV-4 MOR2009/10	0.99	0.95	1.03	1.21	0.96	1.54

a Odds ratio for change of one unit in blood meal *C_T_*; those shown in bold differ significantly (*P *<* *0.05) from 1.

b Odds ratio for membrane feeding compared with feeding on sheep blood; those shown in bold differ significantly (*P *<* *0.05) from 1.

## DISCUSSION

This is the first study to demonstrate the impact of field-derived reassortment on biological transmission of a segmented RNA arbovirus between ruminant and arthropod hosts. Using a highly repeatable and manipulatable *Culicoides*–BTV–sheep model and full genome sequencing data, we show that genome segment inheritance following reassortment in this case was explanatory of phenotypic expression of virus infection and replication in the *Culicoides* vector, while BTV strain pathogenicity following reassortment was unpredictable. These observations have fundamental consequences both for understanding selective pressures operating on reassortment in the field and for the ability to predict the potential for rapid shifts in virus transmission or clinical impact within regions where virus strains circulate.

While tracing a direct lineage between reassortant strains of BTV is challenging, our sequencing data analyses suggested a high degree of similarity in segments inherited from ancestral strains, indicating negligible genetic drift within segment sequences. This inference was aided by a clear timeline in the isolation of strains used for the study and the use of parallel, rather than sequential, comparison of both clinical signs in sheep and infection rates in *Culicoides* that underpinned virus strain characterization. Comparisons of both clinical severity of BTV strain infection in hosts (29, 31, 38) and virus infection rates of vectors (39) have been carried out previously. However, this is the first study to link both these parameters with viruses of known reassortment lineage directly transmitted between sheep and vector *Culicoides*.

Transmission of parental and reassortant strains of BTV from fully infected ITI *Culicoides* to naive sheep was highly efficient across all five strains of BTV, confirming previous studies (40, 41). Interestingly, severity of clinical infection in sheep was not broadly correlated to the number of infective bites received, but, where very few fully infected *C. sonorensis* fed, this occasionally led to a late and slowly progressing RNAemia. During the 2006/7 epidemic of BTV-8 in northern Europe (15), initial outbreaks of disease were classified as being mild, which was initially attributed to reduced exposure to *Culicoides* biting as the incursion began in late summer (42). Our study suggests that a decline in populations of *Culicoides* in autumn is unlikely to change the severity of report cases and that clinical surveillance should still be effective (43, 44).

Rates of viral replication in *C. sonorensis* for four of the five BTV strains were comparable between membrane feeding on viraemic sheep blood and feeding directly from the sheep itself. The exception to this was the BTV-4 MOR2009/07 strain, which produced a significantly higher rate of vector infection in membrane fed individuals. It is unclear at present why this strain-specific variation occurred, as blood meal titers across the testing were similar. Moreover, this result was influenced by a high feeding rate on a single sheep and hence the results should therefore be treated with caution. One potential explanation could be that the RNA quantities were estimated in blood taken from a superficial vein, whereas local virus replication in the skin might lead to local differences from systemic titers (45), leading to variation in vector susceptibility when assessed. More broadly, however, it has been demonstrated here that the systemic level of BTV infection even when determined in blood samples taken at the jugular vein is a valid predictor of vector infection.

No specific pattern was demonstrated in gene segment inheritance for the severity of clinical disease within infected sheep, in part due to the wide variation in manifestation of BT within cohorts. This finding was consistent with previous studies attempting to elucidate the genetic basis of BTV strain virulence, which also could not identify specific genome segments determining virulence in ruminants (32, 33). It is interesting to note that virulence presented as a multi-segmented trait in this study, even when low-passage field isolates were transmitted to the sheep host via blood-feeding *C. sonorensis* infected with BTV. Infection of sheep with BTV-1 MOR 2007/01 did, however, result in a uniform manifestation of what would be considered to be classical clinical BT as described from field observations (7). In contrast, all other strains led to a far more typical intracohort variation in BT severity using the clinical scoring approach. BTV-8 NET 2006/06 was somewhat less severe than expected from previous field reports (12, 13), although this is consistent with previous experiments (16, 31). Anecdotal observation had reported that BTV-4 strains appeared to increase in severity over time following introduction and cocirculation with BTV-1 and BTV-8. While the acute clinical presentation of sheep infected with the early ancestral BTV-4 MOR2004/02 strain was milder in comparison to the two BTV-4 reassortant strains MOR2009/07 and MOR2009/10, this was not reflected in the overall average severity score (Fig. 3 and 6; Table 5), partly through the more chronic presentation of disease in sheep infected with BTV-4 MOR2004/02 still leading to an overall significant clinical impact. Although the pathological damage caused by BTV-4 MOR2004/02 was clearly less severe, this is also compounded by the fact that sheep euthanized during peak clinical disease presented as vastly more acute during postmortem examination compared to those examined later at study end. It is evident, however, that neither of the two BTV-4 reassortant strains inherited the full virulence of its primary ancestral strain BTV-1 MOR2007/01 despite six (BTV-4 MOR2009/07) or four (BTV-4 MOR2009/10) segments originating from this viral lineage.

Infection rates of *Culicoides* fed on viraemic sheep, in contrast to virus virulence, demonstrated consistent differences between viral strains and provided a clear marker of BTV strain characterization during the studies. *Culicoides sonorensis* was almost entirely refractory to two of the ancestral strains (BTV-1 MOR 2007/01 and BTV-8 NET2006/06), while the third ancestral strain (BTV-4 MOR2004/02) resulted in a significantly higher infection rate. This infection rate of *C. sonorensis* was even higher within both of the reassortant progeny (BTV-4 MOR2009/07 and BTV-4 MOR2009/10). The complexity of these trials necessitated the use of colonized *C. sonorensis* to examine virus infectivity and replication ability in the vector. *Culicoides* species from within the Mediterranean and Palearctic region exhibit differential susceptibility for BTV strains according to population and virus strain (46), similar to that observed in the present study.

To investigate the impact of amino acid variation within each viral protein (encoded by the specific genome segment) on virus infection of the vector, we investigated the BTV genomes for amino acid positions that were identical for all three BTV-4 strains, but different for both BTV-1 and BTV-8 parental strains. Seg-7 was the most conserved segment across all five BTV isolates. Four out of five strains shared 100% amino acid similarity in this segment, whereas BTV-4 MOR2004/02 differed by one very conserved amino acid substation (Table 2). Therefore, Seg-7 does not influence the infection rate of these five BTV strains in *C. sonorensis*. Although there were minor amino acid variations in proteins encoded by segments -1 (99.0-99.7%), -3 (99.6-100%), -4 (98.4-100%), -8 (97.7-100%), and -10 (94.7-100%) (Table 2), no single position was identified that was identical for all three BTV-4 strains but different for both BTV-1 and BTV-8. Therefore, these segments also seem to have limited impact on the infection rate of BTV in *C. sonorensis* observed for these strains.

Seg-2, -6, and -9 of the BTV genome, encoding for VP2, VP5, and VP6 respectively, were very similar in amino acid sequence across the three BTV-4 strains that exhibited a high rate of vector infection. For Seg-9, four amino acids substitutions were identified in all three BTV-4 strains, but not in the ancestral BTV-1 MOR2007/01 and BTV-8 NET2006/06 strains. Three of these four amino acid substitutions were classified as conservative and only one as radical (position 95, glycine to arginine). It is unclear if this single amino acid substitution could have any effect on vector competence, but this could be further investigated in the future by generating reverse-engineered mono-reassortant strains.

Most notably, numerous amino acid substitutions have been identified in Seg-2 and -6 between the three BTV-4 strains and the two ancestral strains BTV-1 MOR2007/01 and BTV-8 NET2006/06. It therefore seems probable that VP2 and/or VP5 proteins derived from the parental BTV-4 MOR2004/02 play a key role in determining the vector competence, either individually or in combination, for the BTV strains used in this study. Viral proteins including VP2 from the atypical BTV 26 have been shown to restrict infection and therefore transmission in *Culicoides* (47, 48). The BTV outer capsid protein VP2 is known to be highly variable, containing the epitopes to the host’s neutralizing antibody response, thereby determining the strain serotype (49). Both outer capsid proteins VP2 and VP5 are responsible for cell entry (49), while VP5 also plays a role in the penetration of mammalian and insect cells (50) during the release of the core particle from the endosome. The affinity of VP2 for erythrocyte glycoprotein may facilitate transmission from mammalian blood to the vector (49). Furthermore, VP2 is cleaved by proteases in the saliva of competent vector *Culicoides* resulting in increased infectivity to *Culicoides* derived cells (51). The role of VP2 in the binding, entry, and infection of *Culicoides* mesenteron gut cells, the barrier to infection (52), is as yet undetermined. The ability of BTV-4 MOR2004/04 derived segment -2 (VP 2) to confer the ability of efficient virus replication in *C. sonorensis* has recently been confirmed using reverse-engineered BTV reassortant strains (53).

This study has demonstrated that rapid changes in severity of clinical outcome in the host and likelihood of transmission by vectors can occur, driven by reassortment between cocirculating strains. Reassortants strains may become established even in the presence of the selection and genetic bottlenecks imposed by utilizing both mammalian hosts and insect vectors. The emergence of reassortant strains of BTV possess additional complexity both in the decision to implement vaccination campaigns and in the likelihood of spread in local vector populations as clinical impact and spread by insect transmission may significantly change. Currently, low pathogenicity strains of BTV are often allowed to spread where their impact is perceived as being less damaging than the cost of vaccination (15). In addition, the factors underlying the spread of BTV strains between regions dominated by different species of *Culicoides* are poorly understood, and reassortment could allow these barriers to be overcome.

A future step is to examine the genetic drivers of this process by sequencing of strains exhibiting specific phenotypic characteristics in hosts and vectors. The application of next generation sequencing will be informative in understanding how the virus populations interacting in the process of reassortment are sustained and selected in both the ruminant and insect host. Studies could also be extended to examine tropism of virus communities in both the ruminant and insect host with a view to understanding dissemination. Subsequent testing of viruses produced using reverse genetics and informed by sequencing could also elucidate the genomic basis of a range of phenotypic responses including transmission probability, temperature limits to replication, and pathogenicity in the ruminant host.

## MATERIALS AND METHODS

### Full genome sequencing of BTV strains.

Full genome sequences of BTV strains were obtained by Sanger sequencing (BTV-4 MOR2004/02, BTV-1 MOR2007/01, BTV-4 MOR2009/07, and BTV-4 MOR2009/10), with the exception of BTV-8 NET2006/06, which was additionally resequenced using high throughput sequencing (HTS) (BTV-8 NET2006/06). Sanger sequencing was carried out as previously described (6). For HTS, total RNA was extracted from cell culture pellets using TRIzol Reagent (Life Technologies, Paisley, United Kingdom) and eluted in 100 μL of nuclease free water (Sigma-aldrich, Gillingham, United Kingdom). One microlitre of RNase T1 enzyme was added into each tube and incubated at 37°C for 30 min in a thermocycler to remove ssRNA. DsRNA was purified using the RNA Clean and Concentrator kit (Zymo, Irvine, CA, USA) according to the manufacturer’s recommendations. The purified dsRNA (8 μL) was denatured by heating at 95°C for 5 min and the first cDNA strand synthesized using SuperScript III RT (Life Technologies, Paisley, United Kingdom) while the second strand was synthesized using NEBNext (New England BioLabs, Hitchin, United Kingdom) according to the manufacturers’ instructions. Double stranded cDNA was quantified using the Qubit dsDNA HS assay kit (Life Technologies) and then adjusted to 0.2 ng μL^−1^ with 10 mM Tris-HCl, pH 8.0 buffer. Library preparation was performed using the Nextera XT library preparation kit, and paired end read sequencing (2 ×150 bp) was performed using MiSeq platform and reagent kit v2 (Illumina, San Diego, CA, USA). For sequences obtained by HTS, a prealignment quality check was performed using the FASTQC program v0.11.8, and the Trim Galore script (54) was used for quality and adapter trimming of FASTQ files along with removal of short sequences (<50 bp). Subsequently, reads were mapped to a reference sequence using the BWA-MEM tool (55), and then the DiversiTools software (http://josephhughes.github.io/DiversiTools/; accessed 12 October 2019) was used to generate the consensus sequence. Finally, the consensus sequence was used as a reference sequence to increase the number of BTV reads mapped to the reference, and the final consensus sequence was saved and used for further analysis. GenBank accession numbers for the BTV-8 NET2006/06 strain are MW159097–MW159106.

### Reassortment analysis.

Reassortment analysis was performed using Recombinant Detection Program version 4.95 (RDP4) (56) under default settings. Previously sequenced and published strains were retrieved from GenBank: BTV-1 MOR2007/01 (KP820890, KP821010, KP821132, KP821252, KP821372, KP821492, KP821614, KP821734, KP821855, KP821975), BTV-4 MOR2004/02 (KP820941, KP821061, KP821183, KP821303, KP821423, KP821543, KP821665, KP821785, KP821905, KP822026), BTV-4 MOR2009/07 (KP820942, KP821062, KP821184, KP821304, KP821424, KP821544, KP821666, KP821786, KP821906, KP822027), and BTV-4 MOR2009/20 (KP820945, KP821065, KP821187, KP821307, KP821427, KP821547, KP821669, KP821789, KP821909, KP822030). Multiple sequence alignment was performed separately for each viral segment using the Muscle algorithm in the MEGA6 program (57); coding regions were aligned on the amino acid level. Then, individual alignment files were concatenated using SequenceMatrix software (58) and NEXUS files were used for RDP analysis. The detection of potential recombination events was performed with the following methods: RDP, GENECONV, Maximum Chi Square, CHIMAERA, BOOTSCANing, Sister Scanning (SISCAN) and 3SEQ. Strains BTV-4 MOR2004/02, BTV-1 MOR2007/01, and BTV-8 NET2006/06 were considered as parental sequences based on their year of detection, and BTV-4 MOR2009/07 and BTV-4 MOR2009/10 were investigated as potential reassortant strains.

### Standardization of viral inoculum.

All isolates used were passaged once on *C. sonorensis* derived KC cells to generate infectious tissue culture supernatant (TCS) that was derived in the same cell culture system (Table 1). Briefly a T175 flask of KC cells in suspension of growth media (Schneider’s insect cell media [Sigma-Aldrich, Dorset, United Kingdom]; 10% Gibco heat-inactivated fetal calf serum [ThermoFisher Scientific, MA, USA], and 1% Penicillin/Streptomycin [Sigma-Aldrich, Dorset, UK]) was inoculated with the stock virus from the Orbivirus reference collection and incubated for 7 days at 25°C. Following incubation, cells and supernatants were collected and cells pelleted through centrifugation for 10 min at 1,000 × *g* and 4°C.

The supernatants of each BTV strain were titrated on KC cells using a 96-well endpoint dilution microtitration assay. Ten-fold serial dilutions were added to the titration plates containing 1 × 10^5^ KC cells/well in suspensions of growth media. Titration plates were incubated for 7 days at 25°C at which point supernatants were removed and cells were fixed using 4% paraformaldehyde for 30–45 min, washed 3 × with PBS, and stored under 100 μL PBS/well at 4°C. As KC cells do not develop cytopathic effects, BTV infection of each well was determined through visualization of BTV antigen by immunofluorescence microscopy. Cells were permeabilized with 0.2% Trition for 15 min followed by incubation with anti-BTV guinea pig hyperimmune serum (ORAB279) at 1:2,000 in PBS 0.5% BSA for 1h at room temperature (RT). Following another 3 washes with PBS, plates were incubated for 1h with secondary goat anti-guinea pig-AlexaFluor488 (Invitrogen, United Kingdom) at 1:500 in PBS 0.5% BSA. After 3× final PBS washes, plates were examined under the fluorescence microscope and the presence or absence of fluorescence was recorded for each well.

The final infectious titer for each virus was calculated according to the Spearman-Karber method. Serotype specificity and the absence of cross-contamination between the different virus inocula were confirmed by serotype specific qRT-PCR (59). Viral inoculum characteristics are summarized in Table 1.

### Infection of *Culicoides* with BTV strains.

Colony-derived adults of *Culicoides sonorensis* Wirth & Jones, a BTV vector in North America, were used in the study (60). Maintenance was as described previously (61), with the exception that the colony was sustained using a Hemotek artificial feeder and horse blood from a commercial supplier (TCS Bioscience, Botolph Claydon, United Kingdom). *Culicoides sonorensis* were intrathoracically (IT) inoculated with 0.2 μL BTV tissue culture supernatant at a standardized titer of 5.75 log_10_TCID_50_ for each BTV strain using a pulled glass capillary needle (Narishige, Japan) and a Nanoject II microinjector (Drummond Scientific, PA, USA). Between 50 and 200 *Culicoides* were inoculated for each virus strain in each experimental replicate and were subsequently incubated at 25°C (Binder, Tuttlingen, Germany) in card pillboxes with mesh screen for 5 to 6 days (62). *Culicoides* were given access to 10% sucrose solution on cotton wool that was replenished daily throughout this period.

### Animal experiment.

This animal experiment was carried out in accordance with the UK Animal Scientific Procedure Act (ASPA) 1986, which transposes European Directive 2010/63/EU into UK national law. All animal procedures carried out were reviewed and approved by the Animal Welfare and Ethics Review Board at the Pirbright Institute and conducted in compliance with the relevant project licenses granted by the UK Home Office. The study was conducted under high biological containment at the Pirbright Institute in 4 sequential replicates, each consisting of 6 sheep, with one sheep in each replicate exposed to one parental or reassortant BTV strain, and an associated uninfected control animal (24 sheep total). The sheep used were British mule crosses. All sheep were tested to confirm the absence of anti-BTV antibodies by the UK BTV reference laboratory prior to arrival at The Pirbright Institute using a commercial competitive anti-VP7 antibody ELISA (IDVet, Montpellier, France). All animals were allowed to acclimatize to the new facilities for 6–8 days before onset of any procedures and were fed twice a day with grain pellets and with *ad libitum* access to hay and water throughout the experiment (10).

### Infection of sheep.

Pots containing ITI *Culicoides* were placed on the inner thigh of a restrained sheep to feed for a period of 10 min. The strain of BTV used to infect each sheep and the sheep identification number were recorded. The *Culicoides* were anaesthetized and examined under a light stereomicroscope for evidence of blood feeding. Engorged, or partially engorged, individuals were placed in a microtube containing 100 μL of either Schneider’s media (Experimental Replicates 1 and 2) or RPMI media (Experimental Replicates 3 and 4) to which 2% Penicillin/Streptomycin and Amphotericin B (Sigma-Aldrich, Dorset, United Kingdom) had been added. Samples were homogenized for two cycles of 30 s at 25 Hz with a 3 mm stainless steel bead in a Tissuelyser (Qiagen, Manchester, United Kingdom). The homogenates were then diluted to a total volume of 1,000 μL with RPMI media (Sigma-Aldrich, Dorset, United Kingdom) and 2% antibiotics, the stainless steel bead removed, and samples then vortexed at 13,000 rpm for 10 min and stored at +4°C. Fifty μL of homogenate was then used for nucleic acid extraction and analysis by rtRT-PCR. A C_T_ value of <25 was hypothesized to indicate the that *Culicoides* was likely to have supported a disseminated infection (62) and that transmission of BTV to the sheep was likely to have occurred.

### Monitoring of BTV infection in sheep.

Blood samples were taken from the jugular vein on the day before the experiment began (day 0) and then on days 1, 2, 3, 4, 5, 6, 7, 8, 9, 10, 12, 14, 16, 18, and 19/20 following infection. Clinical signs of disease and rectal temperature were recorded for each animal daily. A postmortem of all sheep was carried out following euthanasia at the study end (19/20 dpi) or when individuals reached a humane endpoint for the protocol.

### Calculation of clinical scores.

A cumulative daily clinical score was determined for each cohort of four sheep infected with a single BTV strain and the four control animals (Table 4). To account for sheep euthanized due to exceeding clinical endpoints, daily accumulated clinical scores for each cohort were divided by the number of sheep present. A total severity score (Fig. 6 and Table 5) for each virus was obtained by dividing the overall accumulated total clinical score from all sheep by the combined days the entire cohort of four individuals stayed alive between 4 and 19 dpi (period for which any clinical signs were recorded).

### Transmission of BTV strains from viraemic sheep to *Culicoides*.

The quantity of BTV RNA in blood samples (RNAemia) taken from sheep in the first 10 days postinfection was tested on the day of collection. RNAemia was used as proxy for viremia in the sheep. Preliminary data suggested that a blood sample with a sqPCR *C_T_* value of less than 25 indicated that BTV infection would reach peak RNAemia in the sheep within the following 2 days, and this was used as a guide to time blood-feeding of *C. sonorensis* (40). *C. sonorensis* were allowed to feed on sheep in card boxes through a fine mesh lid at a density of approximately 250 individuals. The boxes were placed on the inner thigh of a restrained sheep for a period of 10 min, and two boxes of *Culicoides* were fed on each sheep in each replicate. Replete female *C. sonorensis* were selected under light CO_2_ anesthesia using a stereomicroscope. Blood-fed *C. sonorensis* were placed in new card pill boxes and incubated at 25°C with access to a 10% sucrose solution on cotton wool pads that were replenished daily. After an 8-day incubation period postfeeding, all surviving female *Culicoides* were selected under CO_2_ anesthesia and placed individually in microtubes (Qiagen) containing 100 μL Schneider’s media (Experimental Replicates 1 and 2) or RPMI (ThermoFisher Scientific) (Experimental Replicates 3 and 4) for homogenization as described previously. Homogenates were then diluted with 900 μL of either Schneider’s media or RPMI to a total of 1 mL, sealed and stored at +4°C.

### Comparison of feeding methods on infection rate in *Culicoides*.

Blood feeding of *Culicoides* directly on viraemic sheep was complemented by artificial feeding of *C. sonorensis* on sheep blood taken on the matched day of peak RNAemia for each strain in each replicate. Identical boxes of *C. sonorensis* were fed on sheep blood collected the same day as direct feeding. *Culicoides* were fed through a Parafilm (SigmaAldrich, Dorset, United Kingdom) membrane over a reservoir (Hemotek Ltd, United Kingdom) filled with 3 mL of viraemic sheep blood heated to 37°C. Following a period of 30 min exposure to the blood, replete females were selected out under CO_2_ anesthesia and treated as described for the *Culicoides* fed directly on sheep. Any individual *Culicoides* sample with a *C_T_* value <40 when tested by PCR was considered BTV positive. Membrane-fed individuals were exposed to the blood source for a longer period than those fed on sheep to allow for the difference in feeding response to the method, ensuring high feeding rates unconstrained by the need to restrain a sheep for the feeding period. To control for the differential exposure, only fully engorged, replete females were selected for incubation in both feeding scenarios.

### Nucleic acid extraction and semiquantitative sqPCR.

Nucleic acid extraction throughout the studies was carried out using extraction robots. In Replicates 1 and 2, a Universal extraction robot (Qiagen, Germany) and associated nucleic acid extraction kits (Qiagen, Germany) were used. In Replicates 3 and 4, a Kingfisher Flex extraction robot (Thermo Fisher Scientific) and associated kits (MagVet Universal isolation kit) were used. PCR was performed (Replicates 1 and 2) on a Strategene Mx3005P PCR instrument (Agilent, USA) or a Life Systems 7500 Fast PCR instrument (Thermo Fisher Scientific, United Kingdom) (Replicates 3 and 4). Validation of the two molecular diagnostic platforms was carried out to ensure comparable performance, and the PCR assay was ISO/IEC 17025 accredited on both platform systems. A systematic bias of qRT-PCR results due to the change in diagnostic platforms between trials was further controlled within each trial by containing an individual sheep from each viral strain infection group (see above), and no clustering of qRT-PCR results according to trial replicate can be seen in the sheep RNAemia assessment (Fig. 3). In all replicates, a BTV group specific PCR against segment 1 of the virus genome was used (63). Confirmation of BTV serotype of viral stocks was carried out using a serotype specific rRT-PCR assay (59).

In assessments of *Culicoides* infection rates following blood feeding on sheep or through the membrane-based system, 50 μL of pooled homogenate from 8 individually homogenized *Culicoides* was added to a microtube. From this 400 μL pool, nucleic acid was extracted from a 50 μL sample and viral RNA assessed by sqPCR as described above. Individuals contributing to a pooled sample found to contain BTV RNA (≤40 *C_T_*) were subsequently tested individually by sqPCR.

### Statistical analysis.

The effect of strain and number of *Culicoides* feeding at infection on timing and magnitude of peak RNAemia (i.e., the lowest *C_T_* value) was assessed using linear models with time of peak RNAemia or *C_T_* value at peak RNAemia as the response variable and strain and number of *Culicoides* feeding and an interaction between them as explanatory variables. Model selection proceeded by stepwise deletion of nonsignificant (*P > *0.05) terms as judged by *F*-tests.

Vector susceptibility (i.e., the probability of a *Culicoides* midge having a *C_T_* value) after feeding on blood infected with strain *s* by route *f* (either on a sheep [*f *=* *0] or via a membrane [*f *=* *1]) when the *C_T_* value of the infected blood meal is *c* is given by

(1) $$\log\left(\frac{p_{s}(c,f)}{1-p_{s}(c,f)}\right)=b_{0}^{\left(s\right)}+b_{1}^{\left(s\right)}c+b_{2}^{\left(s\right)}f,$$

where the *b_i_*s are strain-specific parameters. Differences among strains were incorporated by assuming hierarchical structure for the model parameters, so that the parameters for strain *s* are drawn from higher-order normal distributions, namely,

$$b_{i}^{\left(s\right)}\sim \text{N}(\mu_{b_{i}},\sigma_{b_{i}}^{2}),$$

where the *μ*s and *σ*s are higher-order parameters.

Parameters were estimated in a Bayesian framework. A Bernoulli likelihood was used for the infection status of each *Culicoides* midge with probability of infection given by equation 1. Priors for the strain-specific parameters were given by the higher-order distributions, while noninformative priors were assumed for the higher-order parameters (diffuse normal for the *μ*s and diffuse gamma for the *σ*s). The methods were implemented in OpenBUGS (version 3.2.3; https://www.mrc-bsu.cam.ac.uk/software/bugs/openbugs/). Two chains each of 10,000 iterations were generated (with the first 2,000 iterations discarded to allow for burn-in of the chains). Convergence of the chains was monitored visually and using the Gelman-Rubin statistic in OpenBUGS.

### Data availability.

GenBank accession numbers for the BTV-8 NET2006/06 strain sequences are MW159097–MW159106.
